# Women’s views and experiences of antenatal care in Iraq: a Q methodology study

**DOI:** 10.1186/1471-2393-14-43

**Published:** 2014-01-23

**Authors:** Nazar P Shabila, Hamdia M Ahmed, Maryam Y Yasin

**Affiliations:** 1 Department of Community Medicine, College of Medicine, Hawler Medical University, Erbil, Iraq; 2 Department of Midwifery, College of Nursing, Hawler Medical University, Erbil, Iraq; 3 Department of Nursing and Midwifery, Erbil Technical Medical Institute, Erbil, Iraq

## Abstract

**Background:**

Understanding women’s experiences and perspectives of antenatal care services is particularly critical for enhancing effectiveness of services delivery and addressing women’s needs and expectations. As part of a comprehensive assessment of the maternity care services in Iraq, this study aimed to explore the views and experiences of antenatal care in a sample of women.

**Methods:**

This explorative study was conducted in Erbil governorate, Iraq. Data were collected using Q methodology, a technique for eliciting subjective views and identifying shared patterns among individuals. A sample of 38 women of different educational and socioeconomic statuses were invited to sort a set of 39 statements reflecting different aspects of the available antenatal care services and issues related to their last pregnancies into a distribution on a scale of nine from “disagree most” to “agree most”. By-person factor analysis was used to derive latent views through centroid factor extraction and varimax rotation of factors.

**Results:**

Analysis of the participants’ Q sorts resulted in identifying four distinct views and experiences of pregnancy and antenatal care services: (i) public maternity services second best: preference for, and ability to afford, private care, (ii) dissatisfaction with public maternity services: poor information sharing and lack of health promotion, (iii) satisfaction with public maternity service but information gaps perceived and (iv) public maternity services second best: preference for private care but unaffordable. The typical characterizations that were associated with each view were highlighted.

**Conclusions:**

This study revealed different patterns of views and experiences of women of pregnancy and antenatal care services and recognized the particular issues related to each pattern. Different patterns and types of problems and concerns related mainly to inadequate provision of information and poor interpersonal communication, poor utilization of public services and a general preference to use private services were identified in the different groups of women.

## Background

Maternal and neonatal mortalities continue to occur at unacceptably high levels in Iraq. The maternal mortality ratio and neonatal mortality rate remained as high as 84 per 100,000 live births and 23 per 1,000 live births, respectively, in 2010 [1]. These figures are significantly higher than developed countries with higher levels of antenatal care. For example the UK had a maternal mortality ratio of 12 and a neonatal mortality of 4 in 2010 [2,3]. Even the neighboring Iran has a considerably lower maternal mortality ratio and neonatal mortality rate than Iraq (30 and 12.5, respectively) [1]. In fact, Iraq is among the group of 68 countries that account for 97% of all maternal and child deaths globally. While neighboring Iran cut its maternal death rate by 220% between 2000 and 2010, Iraq’s rate dropped by a tenth as much during this period. Thus, it is unlikely Iraq can meet its goal of reducing maternal mortality to 20 deaths per 100 000 live births by 2015 [4].

Maternity care services are provided by all levels of the Iraqi health care system. The primary level, which includes a network of public primary health care centers (PHCCs), provides preventive services (antenatal care, growth monitoring and immunization) and curative services (treatment of ailments). Other services provided by some of these PHCCs but to lesser extend include promotion of breastfeeding, family planning and postnatal care [5]. Some of these services particularly curative health services are also provided by the private sector through obstetricians’ private clinics that are widely distributed mainly in urban areas [6].

The Iraqi health care system has been seriously affected as a result of different wars, internal conflicts, international sanctions and political instability during the last few decades [7,8]. These events resulted in a substantial fall in major health indices and left a crippled health system struggling to meet population needs [8,9]. The primary health care system and maternity care services in particular did not escape these damaging effects and continue to suffer from problems common throughout the health care system [6,7,10]. The antenatal care services in Iraq suffer from problems common to the primary health care system. These problems are mainly related to inappropriate health service delivery including irrational use of health services, poor referral system, poor infrastructure, lack of management guidelines and poor hygiene. Other problems include health workforce challenges like poor qualification of health care providers, uneven distribution and rapid turnover of the health workforce and lack of continuing educational and professional development opportunities; and shortage in resources including shortage and low quality of medical supplies and shortage in financing. Poor information technology and poor leadership/governance are also important obstacles to the antenatal care [11,12]. The desperate need for reorganizing and restructuring health services throughout Iraq is increasingly recognized and one of the critical areas that need substantial efforts is maternity care [6,8,13]. Effective restructuring of this important aspect of population health will need a better understanding of its problems, needs and obstacles to its development.

Pregnant women have the rights to participate in decisions involving their well-beings and what may or may not be done to their bodies. Understanding women’s perspectives and experiences of antenatal care services is particularly critical as engagement of women in care is important for enhancing effectiveness of healthcare delivery, giving users a voice and making antenatal care services more responsive to women’s needs and expectations [14,15].

Little research has examined the Iraqi maternity care services particularly from the perspectives of users, i.e. women at reproductive age. As part of a comprehensive assessment of maternity care services, this study aimed to explore the views and experiences of antenatal care in a sample of women. The study results can provide Iraqi policy and decision-makers with information about the quality and utilization of antenatal care services and might assist them in identifying the main access barriers and the potentials for development.

## Methods

### Q Methodology

Q methodology is used to identify different, unique views, as well as commonly shared views. It is particularly useful in research that explores human perceptions and interpersonal relationships [16]. The method allows the researcher to identify groups of participants having similar and alternative viewpoints, and in turn to ascertain similarities and differences among groups. Q methodology effectively combines the strengths of qualitative and quantitative dimensions. In contrast to most qualitative methods, Q data are readily amenable to numerical analyses. Q methodology is explicitly designed to objectively uncover and analyze similarities and differences in the subjective viewpoints of individuals. It is an exploratory, interpretation-intensive methodology suitable for small populations of respondents, and is strengthened by the statistical operation of factor analysis [17,18].

Typically, Q methodology begins with a Q set or a sample of statements that is broadly representative of the contents of the concourse or that offers the fullest range of viewpoints of the study topic [19]. A participant group (referred to as a P set), representing various socio-demographic groups relevant to the study topic, is asked to rank order (Q sort) the Q set along a standardized continuum according to a specified condition of instruction. In contrast to the traditional rating scales that work with absolute responses to statements, Q sorting involves ranking of statements with relative agreement or disagreement where statements only become meaningful in relation to position of other statements. Q analysis involves an inverted factor analytic procedure [20-22]. Correlation between personal profiles indicates similar views, or segments of subjectivity which exist. By correlating people, Q factor analysis gives information about similarities and differences in viewpoint on a particular subject. If each individual would have her/his own specific likes and dislikes, their profiles will not correlate. If, however, significant clusters of correlations exist, they could be factorized, described as common views, and individuals could be measured with respect to them [20].

#### Identification of statements

To determine the issues and views concerning the maternal health services two focus groups were conducted for women who had babies within the past six months and representing different socio-economic groups. The first one involved 12 women from Erbil city center while the second one involved eight women from areas outside Erbil city. A topic guide was used to lead discussions and covered questions on positive aspects of and current problems with the maternity care in addition to the priority needs for its improvement. In addition, five women attending antenatal care facilities and labor wards and two nurses and a gynecologist providing antenatal care services were interviewed. Additional statements were obtained from reviewing relevant literature about the antenatal care services in Iraq [12] and about the viewpoints of women of Pakistani origin in the United Kingdom [23]. The latter study was considered since it is the only identified Q study that assesses the women’s perspectives of maternity services and due to cultural similarities between Pakistani and Kurdish women.

#### Compiling the Q set statements

During the statements identification process, the focus groups and survey participants emphasized mainly on issued related to access to antenatal care facilities, quality of care, interpersonal care and provision of health education, availability of services, the infrastructure of facilities and the role of private sector in providing antenatal care services. As a result of the statement identifications step, 118 statements related to antenatal care were extracted. A structured method was used for refining the statements where statements are selected for different categories and a balanced number of statements were used within the categories [24]. A modified version of the conceptual framework for assessing quality of care that was developed by Donabedian [25] was used to categorize the statements under the main themes of structure, process and outcomes and their specific dimensions of quality assessment. All the statements were reviewed for similarities and differences. Statements that were repeated were discarded, some statements of close similarity were merged and statements that were simply the reverse of other statements were removed.

Two members of the research team made independent decisions about these responses. The aim was to include statements from various aspects of the different themes of Donabedian conceptual framework and their dimensions. The two researchers compared their results and discussed responses which lacked agreement until consensus was reached. The actual expressions of the respondents were used; only the grammar of several statements was edited. Finally, 39 statements that potentially described and sufficiently represented the topic of investigation were selected (Table 1).

**Table 1:** Q set statements and factor array

	**Statement**	**Factor**			
		**1**	**2**	**3**	**4**
1	During my last pregnancy I had regular antenatal care visits in the PHCCs	1	1	2	-2**
2	During my last pregnancy I attended at least one antenatal class	-3	-3	-4	-1**
3	During my antenatal care I got most of my care from one or two people whom I got to know	-1	-1	4**	2**
4	At my antenatal care visits I did not have to wait too long to see the doctor or midwife	0	-2**	3*	1
5	During my last pregnancy I visited more private health facilities and doctors than PHCCs for antenatal care	4**	0	0	-2*
6	I was always treated with respect and kindness during antenatal care visits	2	0	2	0
7	Information about the practicalities of breastfeeding is available during antenatal care	0	-3	-3	1
8	I often used friends and family as sources of information about pregnancy and labor rather than antenatal care services	0*	2	-2*	2
9	The doctors/midwives explained enough about how to recognize the first signs of labor and what to do before going to hospital to have my baby	-1	-4**	-2	0
10	At my antenatal care visits I had enough time talking to either the doctors or midwives	-1	-1	2**	-2
11	Sometimes I was given conflicting advice from the different care providers during antenatal care	-3	-3	-1*	2**
12	There is a good clinical examination at PHCCs for antenatal care	-1	-2	4**	-4**
13	There was a lot of pressure to start a family straight after getting married	-4**	-1	0	-3*
14	Care providers at antenatal care facilities at PHCCs need to have better ‘people skills’	3	3	0*	3
**15**	**The time devoted to patients at antenatal care is adequate**	**0**	**-2**	**-1**	**-3**
16	The women who can afford the expenditure prefer to go to private doctor clinic for antenatal care rather than PHCC	3	3	-2**	4**
17	I have understood the explanations I received from doctors and nurses at antenatal care visits	1**	-1	-1	-2
**18**	**Health expenditures related to pregnancy causes financial burden on my family**	**2**	**1**	**1**	**2**
19	The distance to PHCC is convenient	3**	-1	-4**	-1
20	I usually have seen a nurse rather than a doctor when I went for my antenatal care	1	0	1	3**
21	I complied well with my antenatal care appointment times for follow up	4	2	3	1*
**22**	**The time spent in explaining health status of the woman at antenatal care is appropriate**	**-2**	**-1**	**-1**	**0**
23	The fees for provided antenatal care services (lab tests, ultrasound) at PHCC are convenient	1	1	2	-1**
24	I found it easy to get advice or information when trying to get pregnant	1	1	-3**	2
25	Health education service is provided adequately in health centers	-2	-2	-3	0
26	There are adequacy of antenatal care staffing at PHCCs	-2	0	-2	0
**27**	**Antenatal care providers at health centers are competent and well trained**	**0**	**0**	**0**	**-1**
28	There is a good hygiene situation in the PHCCs	0	2	1	0
29	There are convenient waiting amenities at health centers	-3	1**	-1	-2
30	At private clinic physicians give more time and care to patients than in health centers	2	4*	2	4*
31	At my antenatal care visits I was always encouraged to ask questions	-1	0	-2	1
**32**	**Overall I was very satisfied with the care I received in the antenatal care period**	**-1**	**-2**	**0**	**-1**
33	I would have liked more chance to talk to my care providers for medical advice about care of myself during antenatal care visits	2*	4*	1	1
34	I was subjected to violence from my husband during my pregnancy (physical, psychological, etc)	-4	-4	1	1
35	My last pregnancy was intended pregnancy	1*	3*	-1**	-4**
**36**	**Patient's privacy (and confidentiality) is well respected in PHCCs**	**0**	**1**	**1**	**0**
**37**	**I found out many information about pregnancy myself**	**2**	**2**	**3**	**3**
38	Necessary lab and US investigations are available at PHCCs	-2*	0	0	-3*
39	The waiting time for having services at health centers is appropriate	-2	2*	0	-1

*Distinguishing statement significant at <0.05.

**Distinguishing statement significant at <0.01.

Bold type indicates consensus statement.

#### Creating the Q sort

**Figure 1:**
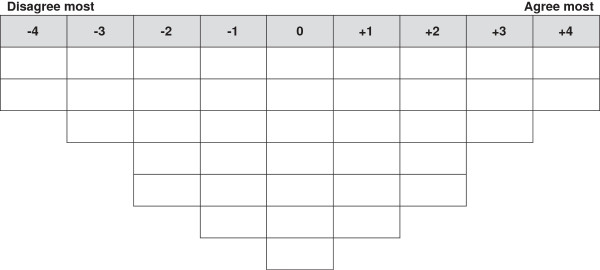
Q sort matrix.

Once the set of statements was confirmed, they were numbered in no particular order to approximate randomization and typed onto small cards with one statement per card. After the Q set was created, the Q sort was developed, which involved creating a quasi-normal distribution with a specific number of cells equal to the number of the Q set statements [20] (Figure 1). This constituted the data collection instrument for the study.

### Participants

The study was carried out on a sample of women from different geographical and socio-economic areas of Erbil governorate from September 2012 to February 2013. Erbil is the capital of Iraqi Kurdistan, a self-ruling region in northern Iraq that is comprised of three governorates out of the 18 governorates of Iraq. Erbil governorate is comprised of eight administrative districts and is inhabited by approximately 1.9 million persons; 75.9% in urban areas and 24.1% in rural areas [26]. There are 12 public hospitals, 197 PHCCs, 7 private hospitals and a large number of private clinics in Erbil governorate [27].

As a general guideline, Q studies consider 40-60 participants to be adequate. Nonetheless, good studies and analysis might easily be carried out with considerably less. Sticking to a number of participants that is less than the number of items in the Q set is ought to be acceptable in most arenas [24]. As the final set of statements was 39 statements, it was decided to select 38 women to participate in the study. Samples in studies employing Q methodology are carefully selected rather than randomized, so that variability in a specific case or situation can be analyzed [28]. Therefore, the study participants were not randomly selected. The sample was purposively selected to include women at reproductive age and having recent experience with antenatal care (i.e. women having a baby during the last year) and representing different age groups and different educational and socio-economic statuses. These women were identified from different geographical locations of Erbil governorate through the help of key informants in those areas.

### Data collection

The selected women were invited to participate in the study at PHCC or private clinic appointments or at their homes. Through a one-to-one session that lasted around 30 minutes the purpose of the study and clear step by step instructions for completing the task were explained to each participant and participant’s consent was obtained. Each participant was asked to sort the cards into 9 piles from -4 (most disagree) to +4 (most agree), in relation to her perception about different aspects of the antenatal care services and issue related to her pregnancy. The researchers recoded the Q sorts on the 9 piles normal distribution grid. After Q sorting, the participants were asked to mention any issues of concern about the antenatal care services and provide any further comments on the subject. These comments were recorded and used in the interpretation of the factors. The study was approved by the Ethics Committee of Hawler Medical University.

### Data analysis

The PQMethod 2.11 program was used for the analysis of Q sorts [29]. The prominent common viewpoints, known as factors, were extracted using centroid factor extraction and varimax rotation. Centroid factor analysis is a way of defining centers of gravity embedded in a correlation matrix. Centroid refers to a kind of grand average of the relationships between all the sorts, because they are represented by their correlation coefficients [30]. Varimax rotation positions factors so that the overall rotated solution accounts for as much of the explained variance as possible. This is achieved by ensuring that each Q sort has a high factor loading on only one factor, an analytic technique that can reveal the majority viewpoints of the sample [24].

Stringent criteria were used for factor selection [31]. Thus, factors representing at least two factor exemplars (i.e. Q sorts or participant responses loading significantly upon one factor alone) and having eigenvalues greater than one were extracted. A conservative significance level of p < 0.01 was chosen for factor loading. Thus, those Q sorts that achieved a factor loading of 0.413 or above on a given factor were considered to have loaded significantly onto that factor [32]. An explanation of how this is calculated is shown Additional file 1. An eigenvalue is the sum of squared loadings for a factor; it conceptually represents the amount of variance accounted for by a factor [33]. However, several different factor solutions were examined for obtaining the most meaningful, consistent and coherent factors. Rotation began with seven factors - those with eigenvalues of above 1.0. Examination of solutions provided with rotation of seven, six and five factors proved unsatisfactory, as the factors produced lacked clarity or definable attributes or resulted in only a single participant loading significantly on certain factor. It was the extraction of four factors that provided the clearest solution, accounting for 45% of the variance in the correlation matrix.

The resultant factors represent sorts that were made by individuals who have responded in essentially the same way. When all of the weighted average scores of the statements of each factor are obtained from the correlation matrix, the statements are arranged in order of descending scores. This arrangement then forms the composite statement array for that factor. To facilitate comparisons between factors, composite statement scores are transformed back into the whole-number scores (+4, +3, etc.) used in the original sorting process. Factor arrays provide a conceptual representation of the factor [34]. Factor interpretation was based upon a thematic reading of statements and their position in the context of all other statements in the final factor arrays [22]. Thus, each factor or view was interpreted subjectively by examining the characterizing and the distinguishing statements. The characterizing statements of a factor are those with a rank value of ‘+4’, ‘+3’, ‘-3’, ‘-4’ in the composite sort. A distinguishing statement for a factor is a statement whose score on that factor is significantly different from its score on any other factor [20]. Distinguishing statements that are significant at p < 0.05 are highlighted with asterisk (*), and those at p < 0.01 are highlighted with double asterisk (**) in the results section. Finally a conceptual interpretation was developed to capture the essence of the viewpoints being endorsed. The comments made by the participants after Q sorting were transcribed and translated to English. Quotations relevant to different themes and subthemes of the extracted factors from the defining participants were derived from these transcripts.

## Results

Thirty eight women participated in the study. Their mean ± SD age was 30.9 ± 6.2 years. Most of them were governmental employees (44.7%) or teachers (21.1%), while 23.7% were housewives. Majority of participants were well educated with only 10.5% having primary education. The participants’ median number of children was 2 (range 1 to 4). Details of the participants’ socio-demographic characteristics are shown in Table 2.

**Table 2:** Socio-demographic characteristics of the participants defining each factor

**Characteristic**	**Factor 1**	**Factor 2**	**Factor 3**	**Factor 4**	Total*
	**(n = 12)**	**(n = 7)**	**(n = 3)**	**(n = 4)**	**(n = 38)**
	**(Count,%)**	**(Count,%)**	**(Count,%)**	**(Count,%)**	**(Count,%)**
**Age (years)**					
20-25	3 (25)	2 (28.6)	-	2 (50)	9 (23.7)
26-30	5 (41.7)	2 (28.6)	-	1 (25)	11 (28.9)
31-40	2 (16.7)	3 (42.9)	2 (66.7)	1 (25)	13 (34.2)
>40	2 (16.7)	-	1 (33.3)	-	5 (13.2)
**Employment status**					
Government employee	5 (41.7)	3 (42.9)	3 (100)	1 (25)	17 (44.7)
House wife	2 (16.7)	4 (57.1)	-	-	9 (23.7)
Student	1 (8.3)	-	-	2 (50)	4 (10.5)
Teacher	4 (33.3)	-	-	1 (25)	8 (21.1)
**Education level**					
Primary school	2 (16.7)	-	-	1 (25)	4 (10.5)
Secondary school	1 (8.3)	4 (57.1)	2 (66.7)		9 (23.7)
Institute**	7 (58.3)	2 (28.6)	-	2 (50)	16 (42.1)
College***	2 (16.7)	1 (14.3)	1 (33.3)		8 (21.1)
Postgraduate	-	-	-	1 (25)	1 (2.6)
**Number of children**					
1	5 (41.7)	1 (14.3)	1 (33.3)	3 (75)	14 (36.8)
2	4 (33.3)	4 (57.1)	1 (33.3)	-	11(28.9)
3	3 (25)	1 (14.3)	-	-	6 (15.8)
4	-	1 (14.3)	1 (33.3)	1 (25)	7 (18.4)

*Including those confounding and non-significant loading.

**Institute; 2 years study after secondary school.

***College; 4 years study after secondary school.

Analysis of the participants’ Q sorts resulted in a four factor solution, i.e. four distinct views and experiences of antenatal care services (Table 1). The four factors were defined by 26 participants (68.4%), whereas seven participants did not have a statistically significant loading on any of the factors and five participants were confounded, i.e. loaded significantly on more than one factor. The socio-demographic characteristics of the participants defining each factor are shown in Table 2. The factor loading for each participant on each of the four factors is shown in Additional file 1.

### Factor 1 – Public maternity services second best: preference for, and ability to afford, private care

Factor 1 is defined by 12 participants. The main characteristic of this group is that they can afford private antenatal care and so us it. Defining participants had some negative experience with the antenatal care services at PHCCs as they complained about lack of convenient waiting amenities (29: -3) and unavailability of laboratory and ultrasound investigations (38: -2*). However, they considered the distance to PHCCs convenient (19: 3**). They wanted to have more chance to talk to care providers during antenatal care visits for medical advice about self care (33: 2*) and thought that care providers at PHCCs need to have better communication skills (14: 3). These participants did not attend antenatal classes at the PHCCs (2: -3). However, they have understood the explanations received from doctors and nurses during antenatal care visits (17: 1**) and have not received conflicting advice from different care providers during antenatal care (11: -3). Example of a quotation from a defining participant representing this theme includes:

There is a need to have better laboratory services and availability of ultrasound in PHCCs. (Participant 05)

Compared to other factors, the defining participants pointed out that they had visited more private health facilities than PHCCs for antenatal care during their last pregnancies (5: 4**) and thought that women usually prefer to go to private doctor’s clinic for antenatal care (16: 3). These participants complied well with antenatal care appointment for follow up during their last pregnancies (21:4). Example of a quotation from a defining participant representing this theme includes:

When I feel worried about my pregnancy, I usually go to see a doctor in the private clinic rather than visiting a PHCC. (Participant 04)

The exemplars of this factor seemed to have self-confidence as they did not experience pressure to start family straight after marriage (13: -4**) and were not subjected to violence from husband during pregnancy (34: -4).

This factor had one neutral distinguishing statement related to using friends and family as source of information about pregnancy and labor rather than antenatal care facilities (8: 0*).

### Factor 2 – Dissatisfaction with public maternity services: poor information sharing and lack of health promotion

Factor 2 has seven defining participants and reflects a general concern about receiving inadequate information and health education about pregnancy and labor during antenatal care visits. This group is characterized by view on how public antenatal care services should change rather than preference for private care. The exemplars defining Factor 2 considered doctors and midwives not explaining enough about how to recognize first signs of labor and what to do before going to hospital to have baby (9: -4**). They liked to have more chance to talk to care providers for medical advice about self-care during antenatal care visits (33: 4*) and thought that care providers at antenatal care facilities at PHCCs need to have better communication skills (14: 3). They thought that physicians at private clinics give more time and care to patients than those at PHCCs (30: 4*). They complained of unavailability of information about practicalities of breast feeding during antenatal care (7: -3) and were unable to attend antenatal classes at PHCCs during last pregnancy (2: -3). Examples of quotations from defining participants representing this theme include:

In some PHCCs nurses do not deal with patients kindly and politely. There is a need to provide better care to patients particularly providing more advice and information. (Participant 35)

Pregnant woman should be better educated about importance of breastfeeding and how and when to breast feed. They should be educated about how to make labor easier like walking, exercise and breathing and care about nutritional values, e.g. not drinking tea after meal. (Participant 31)

However, these participants were not given conflicting advice from different care providers (11: -3), which was the same response as Factor 1.

Exemplars of Factor 2 had a negative experience of waiting long to see doctor or midwife (4: -2**). However, they thought that the waiting time for other services was appropriate (39: 2*) and the waiting amenities were convenient (29: 1**). These women had not experienced violence from husband during pregnancy (34:-4) and their pregnancies were intended (35: 3*). Example of a quotation from a defining participant representing this theme includes:

There is a need for a modern environment for antenatal care services, like not waiting long and to be seen one by one by the doctor. (Participant 30)

### Factor 3 – Satisfaction with public maternity service but information gaps perceived

Factor 3 has three defining participants and emphasizes a positive experience with the antenatal care services although information gaps are perceived.

Exemplars defining Factor 3 complied well with antenatal care appointments for follow up (21: 3) even if the distance to PHCCs was not convenient (19: -4**). They got most of care during antenatal care visits from one or two people whom they got to know (3: 4**). They often used antenatal care as source of information about pregnancy and labor rather than friends and family (8: -2*) and had enough time to talk to the doctor or the midwife (10: 2**). They underlined availability of good clinical examination at PHCCs (12: 4**) and convenient waiting time to seeing the doctor or the midwife (4: 3*). Example of a quotation from a defining participant representing this theme includes:

During antenatal care visits, the doctor and nurses were very kind and cooperative and provided good care to us. (Participant 08)

Compared to other factors’ exemplars, these women seemed to be satisfied with the communication skills of the care providers at antenatal care facilities at PHCCs (14: 0*) and were not given conflicting advice by different care providers (11: -1)*. They did not think that women who can afford expenditure prefer to go to private doctor clinic for antenatal care rather than PHCCs (16: -2**). Example of a quotation from a defining participant representing this theme includes:

I used the PHCC to receive antenatal care as the doctor and nurses were very kind and helpful. (Participant 06)

This group of women had some concerns about provision of information at antenatal care visits as they did not find it easy to get advice or information when trying to get pregnant (24: -3**) and complained of absence of information about practicalities of breast feeding (7: -3). They pointed out to inadequate provision of health education at PHCCs (25: -3) and revealed that they have not attended antenatal classes (2: -4). Example of a quotation from a defining participant representing this theme includes:

During pregnancy I was worried about how to take care of the baby after labor and how to breast feed. (Participant 08)

### Factor 4 – Public maternity services second best: preference for private care but unaffordable

Factor 4 is defined by four participants and represents the women with preference for private antenatal care which is unaffordable to them. This group is characterized by poor utilization of antenatal care that could be due to having unintended pregnancies. Defining participants strongly agreed that they had unintended pregnancy (35: -4**) and did not report any pressure to start family immediately after marriage (13: -3*). They did not have regular antenatal care visits during the last pregnancy (1: -2**) and poorly complied with antenatal care appointments for follow up (21: 1*). They were usually seen by a nurse rather than a doctor during antenatal care visits (20: 3**) and have received most information during pregnancy from one or two persons they got to know (3: 2**). They were sometimes given conflicting advice from different care providers during antenatal care (11: 2**) and were less concerned with attending antenatal care classes (2: -1**). Example of a quotation from a defining participant representing this theme includes:

I visited the PHCC for antenatal care very late and did not have a proper follow up of my last pregnancy. (Participant 02)

These women had negative experience with the antenatal care services at PHCCs as they complained about lack of good clinical examination (12: -4**), unavailability of laboratory and ultrasonic investigations (38: -3*) and poor communication skills of the care providers (14: 3). Example of a quotation from a defining participant representing this theme includes:

The nurses are usually very busy and are not very keen to talk to us. (Participant 18)

These women showed a general preference for visiting private health facilities for antenatal care if they can afford expenditure (16: 4**) and thought that physicians at private clinics give more time and care than those at PHCCs (30: 4*). However, they did not visited private facilities more than PHCCs during last pregnancy (5: -2*) and had concerns about the high fees of antenatal care provision at PHCCs (23: -1**).

### Consensus statements

There were seven statements of consensus with no statistically significant difference in scores across the four factors. The consensus was mainly around an overall dissatisfaction with the antenatal care services (32: -2 to 0), inadequacy of time devoted to patients (15: -3 to 0) and time spent on explaining health status of the women (22: -2 to 0), self-dependant to find information about pregnancy (37: 2 to 3) and the high pregnancy-related health expenditures (18: 1 to 2).

The other two statements showed neutrality around considering the antenatal care providers at PHCCs competent and well trained (27: -1 to 0) and respecting patient’s privacy and confidentially in PHCCs (36: 0 to 1).

## Discussion

This explorative study revealed different patterns of views and experiences of women of the antenatal care services in Erbil governorate. One positive and three generally negative patterns of women’s views and experiences of the antenatal care services in the public sector were identified. The variety in women’s views and experiences of antenatal care might be attributed to different factors related to health facility infrastructure and the available facilities, quality of services, qualifications and experiences of health care providers and/or specific experiences, expectations and characteristics of women. Reporting presence of differences in views and experiences of women concerning antenatal care in Iraq might not be totally new knowledge by itself. However, this study was able to identify and characterize these differences in a novel and insightful way.

Respondents loading on Factor 1 reflected a typical model of health seeking behavior that result from high dissatisfaction or negative experience with the available public services that eventually lead to the use of private services [35]. The private health services in Iraq are widely used particularly by affluent people since these services are widely distributed, primarily in urban areas, and are easily accessible [36]. However, women cannot completely rely on private services for antenatal care as some services like immunization are only available in the public sector. These women seemed to have a better experience because they could afford to use the more expensive private care.

The main concerns of respondents loading on Factor 2 were related to poor provision of information about pregnancy and labor during antenatal care visits. Antenatal care is usually viewed as an important point of contact between care providers and women and an opportunity for provision of health education including how to detect pregnancy complications and how to develop a birth plan [37]. Exemplars of the other three factors, particularly those of Factor 3, had also concerns about inadequate provision of information and poor interpersonal communication.

In general, health education and provision of information to women at antenatal care are not formally defined as job description of nurses and midwives in Iraq. Another study from Iraq similarly reported poor provision of health education at antenatal care units in PHCCs in Erbil city as only 23.7% of visiting women received education about care for the baby and breast feeding, 53% were told about progress of pregnancy, 55% had the chance to ask questions and 65% were asked to return for another visit [12]. A study from Duhok governorate of Iraqi Kurdistan region also reported poor provision of health education in the PHCCs and peoples’ poor perception and understanding of the existing health education messages [38]. In neighboring Jordan, a study reported poor use of health education sessions at PHCCs by clients and the clients not usually recommending these sessions to others as the sessions were not meeting the specific health education needs of the clients [39]. A study from Iran revealed that clients’ satisfaction was lowest with provision of health education at PHCCs than other primary care services [40]. A study from Egypt also reported poor satisfaction (<30%) with health education methods at antenatal care services and with the explanation of the problems by physicians [41].

The women loading on Factor 3 looked to take a good care of their pregnancies and had a positive experience with antenatal care services at PHCCs particularly in terms of access and quality of care. They clearly preferred to use the antenatal care services at PHCCs rather than private services. The financial situation had no effect on utilization of private facilities. Another study from Iraq also revealed a satisfactory average number of antenatal visits, but there was poor early booking at health centre [42]. A study from Egypt similarly reported a high clients’ satisfaction with the quality of antenatal care services [41].

Although the women loading on Factor 4 had negative experience with the antenatal care at PHCCs and preferred to use private care, they rarely used the latter. This could be the kind of viewpoint of many poor people who cannot afford to use the private care particularly that this group had concerns about the high fees for provided services even at PHCCs. Women’s poor perception of antenatal care at PHCCs could be due to having unintended pregnancy that might have resulted in poor utilization of antenatal care services. Significant association between having unintended pregnancies and poor antenatal care or late antenatal care initiation has been reported in different settings like Tanzania and Ecuador [43,44].

There was a consensus among exemplars of three of the four groups about a general dissatisfaction with antenatal care services; although antenatal care together with immunization have been recognized as the best functioning services in PHCCs in Iraq [45]. These preventive services have better financial, logistical and training support in comparison to other programs [46]. Recognition of these two services as best functioning services does not mean that they are working perfectly and do not suffer from shortcomings. Studies from Afghanistan and Nigeria have also reported a generally better satisfaction with maternal and child care services in PHCCs compared to other services [47,48]. There was also a general consensus about devoting inadequate time to patients at antenatal care. Spending inadequate time with care providers has also been reported by another study from Iraq where 61% of women spent three minutes or less with the health care provider in the PHCCs in Erbil city [12]. All factors emphasized the financial burden resulting from pregnancy and labor. Difficulty to afford health care in Iraq was also reported by a household survey where 16.9% of households had to borrow money and 7.4% had to sell assets in the month preceding the study to meet health costs [36].

Poor access, coverage, utilization and quality of antenatal care services that were reported by the different groups of women in this study are also common problems in many developing countries [49]. In contrary, research has shown that women in developed countries like Canada are generally satisfied with the antenatal care services where clinical and interpersonal care processes in addition to certain aspects of structure are considered most essential to quality of care [15].

This study used Q methodology to assess women’s perspectives and experiences of the antenatal care services in Erbil, Iraq. By employing Q methodology, this study was helpful in understanding the women’s utilization of antenatal care and their health seeking behavior in relation with their perception of the available services. Through combining the strengths of both qualitative and quantitative research, Q methodology was of added value in this respect as mixed quantitative and qualitative methods have been recommended to help in capturing both prevalence of behaviors according to specific health conditions and the rationale for specific health seeking behavior pathways [50,51]. This study can direct future quantitative and qualitative research in this field. For example, presentation of the identified views on the basis of the characterizing and distinguishing statements to a representative sample of women could determine different socio-demographic factors associated with each view. In-depth qualitative studies can explore different social and cultural factors responsible for the uncovered views.

This Q study has also some specific potential limitations. The views and experiences of women from lower socio-economic groups might have been overlooked as administration of Q sort requires the respondent to have a certain level of education. Typically, the Q statements in the same Q set should be of the same attitudinal type that can be sorted on the Likert type scale used from ‘disagree most’ to ‘agree most’ on the 9 point scale [52]. However, the Q set of this study contained a number of factual items that are of yes/no response (e.g. items 2, 20, 34 and 35). Presence of these items can cause measurement problems partly because two types of item are mixed up making it difficult for the respondent to rank statements, but mostly because one can’t score responses to a factual item in the same way as an attitudinal one on the grid. This could have a negative impact on interpretation of the factors. For example, the item on spousal violence is ranked ‘-4’ in Factor 1 and Factor 2 suggesting most if not all women here did not experience it, whereas it is ranked ‘1’ in Factor 3 and Factor 4 suggesting the responses were averaged with some did and some did not experience it. While intimate partner violence against women is common in Iraqi Kurdistan region [53,54], the question of spousal violence could not help to determine its effect on women’s experience with pregnancy and antenatal care. Factual items like this would have been better asked as part of a survey and used to contextualize the factor interpretation.

## Conclusion

This study revealed different patterns of views and experiences of women of the antenatal care services and recognized the particular issues related to each pattern. Different types of problems and concerns related mainly to inadequate provision of information and poor interpersonal communication, poor utilization of public services and a general preference to use private services were reported by the different groups of women. Awareness of the health policy makers and maternity care managers of these problems and concerns might direct action to improve these services.

## Abbreviations

PHCC: Primary health care center.

## Competing interests

The authors declare that they have no competing interests.

## Authors’ contributions

NPS, HMA conceptualized and designed the study. HMA and MYY collected the data. NPS carried out data analysis and interpretation. NPS and HMA prepared the manuscript. MYY and HMA extensively reviewed and edited the manuscript. All authors read and approved the final manuscript.

## Pre-publication history

The pre-publication history for this paper can be accessed here:

http://www.biomedcentral.com/1471-2393/14/43/prepub

## Supplementary Material

Additional file 1 Participants’ characteristics and factor loading on the four factors. Click here for file
