# Supplementary material for: Women’s views and experiences of antenatal care in Iraq: a Q methodology study
Source: BMC Pregnancy Childbirth. 2014 Jan 23;14:43. doi: 10.1186/1471-2393-14-43 (PMC3902000; doi:10.1186/1471-2393-14-43)
Supplement: Additional file 1 — Participants’ characteristics and factor loading on the four factors. [file 1471-2393-14-43-S1.pdf]

Additional files

Additional file 1 – Participants' characteristics and factor loading on the four factors

Participant No.	Occupation	Factor loading			
		**1**	**2**	**3**	**4**
1	Teacher	**0.581**[x]	-0.171	0.231	0.149
2	Government employee	0.088	0.133	-0.041	**0.449**[x]
3	Government employee	0.071	0.373	0.116	0.256
4	Housewife	**0.603**[x]	0.086	0.008	0.246
5	Government employee	**0.685**[x]	0.314	0.188	0.303
6	Government employee	0.127	0.155	**0.794**[x]	0.073
7	Government employee	0.031	0.046	**0.891**[x]	-0.102
8	Government employee	0.159	0.185	**0.848**[x]	0.001
9	Teacher	**0.567**	**0.454**	0.153	-0.027
10	Teacher	**0.572**[x]	0.306	0.093	-0.091
11	Government employee	-0.232	0.269	0.134	-0.272
12	Teacher	**0.776**[x]	0.204	-0.004	-0.011
13	Government employee	0.038	0.336	-0.111	0.066
14	Government employee	**0.592**[x]	0.166	-0.009	-0.025
15	Government employee	**0.420**[x]	0.020	0.038	-0.032
16	Student	0.395	0.040	-0.100	**-0.481**[x]
17	Student	**0.593**[x]	0.303	-0.11	0.056
18	Student	0.333	0.282	0.073	**0.434**[x]
19	Housewife	0.003	-0.052	0.009	-0.271
20	Teacher	0.364	0.142	0.219	**0.446**[x]
21	Teacher	**0.578**[x]	0.37	0.246	0.173
22	Government employee	0.156	**0.441**[x]	0.077	0.238
23	Housewife	0.325	-0.324	0.253	-0.362
24	Government employee	**0.484**[x]	0.406	0.224	0.196
25	Teacher	0.305	0.116	0.267	0.247
26	Teacher	0.401	0.259	-0.229	-0.172
27	Housewife	0.375	**0.688**[x]	0.235	-0.094
28	Government employee	**0.425**	**0.582**	-0.005	0.167
29	Housewife	0.333	**0.783**[x]	0.147	0.128
30	Housewife	0.404	**0.603**[x]	0.333	-0.011
31	Housewife	0.322	**0.585**[x]	-0.073	0.178
32	Housewife	**0.633**[x]	0.15	0.077	0.293
33	Government employee	0.361	**0.772**[x]	-0.075	0.114
34	Student	-0.002	-0.083	0.335	0.036
35	Government employee	-0.032	**0.486**[x]	-0.003	0.069

36	Government employee	**0.591**	0.36	0.163	**0.456**
37	Housewife	**0.433**	**0.428**	0.123	0.103
38	Government employee	**0.573**^{x}	0.362	0.181	0.005
Eigenvalues		10.90	2.03	2.28	1.93
Defining sorts		12	7	3	4
Explained variance %		29	5	6	5
Explained variance cumulative %		29	34	40	45

Bold type indicates significant loadings. Significance at the 1% level is taken as a factor loading greater than (2.58 x 1√n), where n= the number of statements - so in this case significant loadings are those higher than 0.413.

^{x} indicates defining sorts.
